# Identification and characterization of laccase-type multicopper oxidases involved in dye-decolorization by the fungus *Leptosphaerulina* sp.

**DOI:** 10.1186/s12896-015-0192-2

**Published:** 2015-08-14

**Authors:** Ledys S. Copete, Xiomara Chanagá, Jorge Barriuso, María F. López-Lucendo, María J. Martínez, Susana Camarero

**Affiliations:** Centro de Investigaciones Biológicas, CSIC. Ramiro de Maeztu 9, Madrid, 28040 Spain; PROBIOM, Universidad Nacional de Colombia Sede Medellín, Calle 59ª No 63-20, Medellín, Colombia; Present address: Universidad Manuela Beltrán, Calle de los Estudiantes 10-20, Bucaramanga, Colombia

**Keywords:** *Leptosphaerulina*, Multicopper-oxidases, Laccases, Phylogeny, Secretome, Dye decolorization, Natural mediators

## Abstract

**Background:**

Fungal laccases are multicopper oxidases (MCOs) with high biotechnological potential due to their capability to oxidize a wide range of aromatic contaminants using oxygen from the air. Albeit the numerous laccase-like genes described in ascomycete fungi, ascomycete laccases have been less thoroughly studied than white-rot basidiomycetous laccases. A variety of MCO genes has recently been discovered in plant pathogenic ascomycete fungi, however little is known about the presence and function of laccases in these fungi or their potential use as biocatalysts. We aim here to identify the laccase-type oxidoreductases that might be involved in the decolorization of dyes by *Leptosphaerulina* sp. and to characterize them as potential biotechnological tools.

**Results:**

A *Leptosphaerulina* fungal strain, isolated from lignocellulosic material in Colombia, produces laccase as the main ligninolytic oxidoreductase activity during decolorization of synthetic organic dyes. Four laccase-type MCO genes were partially amplified from the genomic DNA using degenerate primers based on laccase-specific signature sequences. The phylogenetic analysis showed the clustering of Lac1, Lac4 and Lac3 with ascomycete laccases, whereas Lac2 grouped with fungal ferroxidases (together with other hypothetical laccases). Lac3, the main laccase produced by *Leptosphaerulina* sp. in dye decolorizing and laccase-induced cultures (according to the shotgun analysis of both secretomes) was purified and characterized in this study. It is a *sensu-stricto* laccase able to decolorize synthetic organic dyes with high efficiency particularly in the presence of natural mediator compounds.

**Conclusions:**

The searching for laccase-type MCOs in ascomycetous families where their presence is poorly known, might provide a source of biocatalysts with potential biotechnological interest and shed light on their role in the fungus. The information provided by the use of genomic and proteomic tools must be combined with the biochemical evaluation of the enzyme to prove its catalytic activity and applicability potential.

**Electronic supplementary material:**

The online version of this article (doi:10.1186/s12896-015-0192-2) contains supplementary material, which is available to authorized users.

## Background

Multicopper oxidases (MCOs) are a multifaceted group of proteins with diverse functions, ranging from copper and iron metabolism (e.g. ceruloplasmin) to polyphenol oxidation (e.g. laccases), and different domain folding. Among MCOs, laccases, ascorbate oxidases, ferroxidases, bilirubin oxidases and pigment oxidases, organized in three cupredoxin-like domains, constitute the laccase-type group [1]. MCOs catalyze the oxidation of a wide variety of substrates with the concomitant reduction of molecular oxygen to water. There are different coordination modes for copper in MCOs: the Type 1 (T1), Type 2 (T2), and Type 3 (T3) copper centers that differ on their spectroscopic and EPR properties. In a typical MCO oxidation, the abstraction of four separate electrons takes place at the T1 copper site. Then, fast intramolecular electron transfer occurs from T1 site to the trinuclear Cu cluster (one T2 and two T3 copper ions) where oxygen binds and is converted to water.

Laccases typically show phenol oxidase activity. Besides, they oxidize a wide range of substituted aromatic amines, N-heterocycles, phenothiazines, thiol groups, etc. Laccases are found in vascular plants, fungi, bacteria, and insects, although the wood rotting and litter decomposing basidiomycete fungi are the main laccase producers. Laccases from white-rot fungi have been widely studied and characterized due to their role in lignin biodegradation. On the contrary, only a few ascomycete laccases (e.g. from *Myceliophthora thermophila, Melanocarpus albomyces, Thielavia arenaria* or *Neurospora crassa*) have been fully characterized, even though a number of laccase-like genes have been described in numerous ascomycetes so far [2, 3]. Fungal laccases are supposed to be involved in various physiological functions such as fruiting body development and pigmentation, copper homeostasis, or pathogenesis [4–6] in addition to their function in lignin degradation and plant litter decay processes [7, 8]. Nevertheless, further research effort is needed to assign concrete biological functions to the multiplicity of laccase-type MCOs of a fungal species.

The *Dothideomycetes* class of ascomycetes includes many important plant pathogens affecting all major crop plant families although *Leptosphaerulina spp* have been also described as saprophytes [9]. A variety of MCO genes has recently been discovered by the genome sequencing of different dothideomycetes fungi from the *Pleosporales* order: *Leptosphaeria maculans* which infects the oilseed rape [10], *Pyrenophora tritici-repentis*, a necrotrophic fungus causal to the disease tan spot of wheat (http://www.broadinstitute.org) and *Phaeosphaeria (Stagonospora) nodorum* which is also a pathogen of wheat (http://www.broadinstitute.org). However, little is known about the presence and function of laccases in the *Dothideomycetes* class or their potential use as biocatalysts.

Recently, an ascomycete strain from the *Pleosporales* order, isolated from lignocellulosic material in the Aburra Valley (Antioquia, Colombia), was selected due to its capability to decolorize different synthetic organic dyes. The identification of the fungus as *Leptosphaerulina* sp. was based on the sequence analysis of ITS1-ITS2 regions and 28S rDNA and on morphological characteristics. The strain grown in malt extract plates showed ABTS oxidation activity, and laccase activity was detected as well during fungal decolorization of Reactive Black 5 in liquid cultures [11]. The sole information reported in years about the presence of laccases in *Leptosphaerulina* spp. dates back to 1979, when laccase was localized in the hyphae cell wall of *Leptosphaerulina briosiana* [12]. Recently, a laccase from *Leptosphaerulina chartarum* has been characterized as well [13].

We aim here to identify the laccase-type MCOs of this *Leptosphaerulina* strain by combing the use of genomic and proteomic tools and to characterize, by classical biochemistry methodologies, the laccases possibly involved in dye decolorization that could be useful as potential biotechnological tools.

## Methods

### Microorganism and culture conditions

*Leptosphaerulina* sp. CECT (20913) was isolated from lignocellulosic material in the Aburrá Valley (Colombia). The fungus was maintained in 2 % malt extract agar. Liquid cultures were carried out in 1 L-flasks with 250 mL of medium containing glucose, ammonium tartrate, peptone and yeast extract [14]. When necessary the medium was supplemented with 200 mg/L of the azo dye Reactive Black 5; or with 500 μM CuSO_4_ and 9 g/L ethanol as laccase inducers (laccase-inducing culture). Inocula consisted of 5 mL of mycelium grown for 15 d in malt extract under stationary conditions and homogenized in a Sorvall Omni-Mixer at 16,000 rpm for 30 s. Flasks were incubated at 25 °C and 200 rev min^−1^ and the ligninolytic oxidoreductase activities secreted by *Leptosphaerulina* sp. were monitored for 16 days.

### Enzymatic assays

The oxidation of 3 mM ABTS (2,2-azino-bis(3-ethylbenzothiazoline-6-sulfonic acid) was followed the formation of the radical cation (ε_418_ = 36,000 M^−1^ cm^−1^) in 100 mM sodium tartrate buffer pH 3, in the absence or presence of 0.1 mM H_2_O_2_ to determine laccase and peroxidase activities, respectively. Mn^2+^ oxidation was followed at pH 5 for the formation of Mn^3+^ tartrate complex (ε_238_ = 6,500 M^−1^ cm^−1^). Dye oxidation were assayed at pH 3 by monitoring the disappearance of 50 μM Reactive Black 5 (ε_598_ = 30,000 M^−1^ cm^−1^) and 25 μM Reactive Blue 19 (ε_595_ = 10,000 M^−1^ cm^−1^). Reactions were initiated by the addition of 0.1 mM H_2_O_2_. One enzymatic activity unit was defined as the amount of enzyme that oxidizes 1 μmol of substrate in 1 min.

### Production and purification of laccase

The supernatant of a three-day culture supplemented with 500 μm CuSO_4_ and 9 g/l ethanol, was centrifuged, filtered and concentrated by ultra filtration using a 5-kDa cut-off membrane. The concentrated sample was precipitated with sulphate ammonium (85 %). The precipitate was centrifuged, dissolved in 20 mM Bis-Tris buffer, pH 6.5 and concentrated (Amicon 10-kDa-cut-off). The sample was then subjected to anionic-exchange chromatography using a Hi Trap Q FF 5 mL column (GE Healthcare) pre-equilibrated with 20 mM Bis-Tris, pH 6.5, and connected to an AKTA Purifier System (Amersham Biosciences). Proteins were eluted with a 0 – 25 % NaC 1 M gradient, at 1.5 ml/min flow rate. Fractions with laccase activity were pooled, dialysed, concentrated and subjected to high-performance anion-exchange chromatography using a Mono Q 5/50 GL column (GE Healthcare) equilibrated with the same buffer. The retained proteins were eluted with a 0 – 6 % NaCl 1 M gradient, at 0.5 ml/min, and laccase fractions were pooled, dialysed and concentrated. The last purification step consisted of size-exclusion chromatography with a Superdex 75 FPLC column (Pharmacia). The protein was eluted with 0.15 M NaCl at 0.3 mL/min, dialysed and concentrated in Amicon Ultra-15 (Millipore).

### Laccase characterization

Pure laccase (6 μg of protein) was deglycosylated with 15 mU of endo-β-N-acetylglucosaminidase H (Endo-H) in 50 mM sodium tartrate buffer, pH 5.5, at 37 °C overnight. Polyacrylamide electrophoresis under denaturizing conditions (SDS-PAGE) was performed at 7.5 % (p/v) and the protein bands stained with Coomassie Brilliant Blue. Laccase molecular mass was determined before and after deglycosylation by MALDI/TOF-TOF (Matrix-assisted laser desorption/ionization-time of flight). Isoelectric point was determined by isoelectrofocusing electrophoresis with Criterion IEF Precast gel (Bio-Rad) using a pH range from 3 to 10. The UV-visible absorption spectrum of the enzyme was recorded in a Shimadzu spectrophotometer.

Kinetics for the oxidation of ABTS (ε_418_ = 36,000 M^−1^ cm^−1^) and 2,6-dimethoxyphenol (ε_469_ = 27,500 M^−1^ cm^−1^) were determined at 25 °C, in 0.1 M sodium tartrate buffer pH 3, using concentrations between 0 and 1 mM ABTS and 0 – 5 mM DMP. The kinetic constants were calculated from Michaelis-Menten equation in Sigma Plot Software. Optimum pH for laccase activity with ABTS and DMP was determined within a pH range of 2 –9 in 0.1 M Britton and Robinson buffer. The stability of the enzyme against pH was calculated by the difference between the initial laccase activity and the activity remaining after 180 min of incubation in 0.1 M Robinson buffer at different pH (2 – 9). The thermal stability of the purified laccase was determined in a temperature range of 30 – 80 °C, for 10 min, in 0.1 M sodium tartrate buffer pH 3 with ABTS as substrate. All measurements were made in triplicate in a Versa Max Microplate Reader (Molecular Devices).

### Dye decolorization assays

Enzymatic decolorization of synthetic organic dyes was assayed with 100 mU of crude *Leptosphaerulina* sp. laccase in 0.1 M sodium tartrate buffer pH 3, at room temperature. The reaction mixture contained 50 μM dye (except for Remazol Brillant Blue that was 100 μM) and, when necessary, double concentration of the following mediators: acetosyringone, syringaldehyde, methyl syringate or 1-hydroxybenzotriazole. Controls without enzyme were carried out in parallel. All measurements were performed in triplicate, after 0, 2, 3 and 4 hours, in the microplate reader. Dye decolorization was expressed in terms of percentage of the absorbance decrease at the maximum wavelength for each dye: Methyl Orange (azo-dye), 500 nm; Reactive Black 5 (di-azo), 597 nm; Evans Blue (di-azo dye), 605 nm; Orange II (azo-dye), 484 nm; Remazol Brilliant Blue (anthraquinone), 595 nm; Acid Blue 74 (indigo), 609 nm; and Aniline Blue (triphenylmethane), 583 nm.

### Trypsin digestion and peptide analysis of the pure protein

Pure laccase was digested with trypsin and the resulting peptides were analyzed by an LTQ-Orbitrap Velos mass spectrometer coupled to a nano Easy high-performance liquid chromatography (Thermo Sccientific). Peptides were trapped onto a C18-A1 2 cm precolumn and then eluted onto a Biosphere C18 capillary column using solutions A and B (0.1 % formic acid in pure acetonitrile) at a flow-rate of 250 nL/min with the following gradient: 0-35 % Buffer B, for 55 min and 35-45 % Buffer B, for 10 min. Full-scan MS spectra (m/z 300–1700) were acquired in the positive ion mode. The 15 most intense ions were selected for collision induced dissociation (CID) fragmentation. Mass spectra files were searched against Uniprot database (http://www.uniprot.org/) and The Laccase and multicopper oxidase Engineering Database (LccED, http://www.lcced.uni-stuttgart.de/cgi-bin/LccED1.2/index.pl) using SEQUEST through Proteome Discoverer (version 1.4.1.14).

### Amplification of laccase-like genes

Genomic DNA was extracted from fungal mycelium grown in malt extract liquid medium using DNeasy Plant Mini Kit (Qiagen). Laccase-like genes were amplified using degenerate primers based on the sequences of the conserved copper binding motifs L2 (HXH) and L3 (HXXHXH) of laccases from related ascomycete fungi (LAC2FOR 5’- GGIACIWIITGGTAYCAYWSICA -3’ and LacL3Rv 5’- GTCGTGKCCGTGSARRTGGA -3’). A degenerate primer (Fw 5’- GCWAAATGGGGYGACACKATY -3’) based on the 7 first amino acid residues of the peptide found in the proteomic analysis of the pure enzyme (AKWGDTI) was also used for the amplification of *Leptosphaerulina* sp. laccase genes from the genomic DNA. The amplification reactions contained 100 ng of DNA template, 1.5 mM MgCl_2_, 0.8 mM dNTPs, 0.5 μM of each primer and 1 U de Taq DNA polymerase (Invitrogen) in 50 μL final volume. The amplification program was as follows: 3 min at 95 °C; 30 cycles of 45 s at 95 °C, followed by 45 s at 52 °C and 2 min at 72 °C; and a extension step for 5 min at 72 °C. PCR products were purified with the QIAquick Gel Extration Kit. The amplified sequences were cloned into pGEM-T easy cloning system (Promega) and transformed into *Escherichia coli* DH5α cells. Clones containing the inserted fragments were screened by DNA sequencing using the BigDye Terminator v3.1 Cycle Sequencing kit. The nucleotide sequences were translated and the introns (one in *lac3* from nucleotide 178 to 228, and other in *lac2* from nucleotide 133 to 180) were removed.

### Phylogenetic analysis

Phylogenetic analysis for the four deduced amino acid sequences of *Leptosphaerulina* sp laccase-like proteins was based on MUSCLE multiple alignment with partial protein sequences (L2 to L3 length) from different types of fungal MCOs (basidiomycete laccases, ascomycete laccases, ascorbate oxidases, ferroxidases, fungal pigment oxidases). The evolutionary history was inferred using the Neighbor-Joining method. Bootstrapping was carried out with 1000 replications. The evolutionary distances were computed using the JTT matrix-based method. All positions containing gaps and missing data were eliminated. Evolutionary analyses were conducted in MEGA6. The tree was rooted by two insect laccase sequences.

### Secretome analysis and peptide analysis

Crude extracts from liquid cultures supplemented with RB5 (after 7 days of incubation) or with copper and ethanol as laccase inducers (after 3 days of incubation) were filtered through 0.22 μm membranes and concentrated by tangential ultrafiltration (Amicon 5-kDa-cut-off).

Sample impurities were removed by short PAGE stained with Colloidal Blue. Samples of around 5 μg of protein [15] were dissolved in sample buffer (37.5 mM Tris–HCl pH 8, 1.5 % (w/v) SDS, 1 mM EDTA, 1.96 mM DTT, 0.005 % (w/v) bromophenol blue and 12.5 % (v/v) glycerol) and run into a 12 % SDS-gel. Excised bands were distained with 50 mM ammonium bicarbonate, dehydrated with 50 % ACN and dried. The dried gel pieces were reduced with 10 mM dithiothreitol for 30 min at 56 °C, alkylated with 55 mM iodoacetamide in obscurity for 30 min (24 °C) and digested with 12.5 ng/μL trypsin in 50 mm ammonium bicarbonate, overnight at 30 °C. Peptides were extracted at 37 °C using 100 % ACN and then 0.5 % trifluoroacetic acid, dried, cleaned using ZipTip with 0.6 μL C18 resin (Millipore).

The tryptic peptides were reconstituted in 5 μL solution A (0.1 % formic acid in 2 % acetonitrile), and analyzed in the LTQ-Orbitrap Velos. The peptides from EPP digestions were eluted with a 0-45 % Buffer B gradient, 210 min. Full-scan MS spectra (m/z 300–1800) were acquired as aforementioned. Mass spectra files were searched against Uniprot and LccED databases as described above. Search parameters included a maximum of two missed cleavages allowed, carbamidomethylation of cysteines as a fixed modification and oxidation of methionine as variable modifications. Identified peptides were validated using Percolator algorithm [16]. Besides, an in-house database was constructed with the partial laccase-type sequences of *Lepthosphaerulina* sp. disclosed in this study (Lac1-Lac4), to investigate the presence of these MCOs among the oxidoreductases secreted by the fungus under dye-decolorizing and laccase-inducing conditions.

## Results

### Characterization of ***Leptosphaerulina*** sp. laccase

*Leptosphaerulina* sp. was grown in liquid medium in the absence and in the presence of the azo dye Reactive Black 5. The fungus initiated dye decolorization in the third day of incubation, coinciding with the maximum laccase activity (260 U/L). The dye was decolorized up to 95 % within 7 days. Laccase levels rose in the dye-decolorizing culture as compared to the maximum activity found in the standard culture (175 U/L after 5d). A generic peroxidase activity with ABTS was also detected in the liquid cultures (maximum activity in 7–9 days), with similar activity values in the absence or presence of the dye (Additional file 1: Figure S1). By contrast, lack or very low ligninolytic peroxidase activities (<1 U/L) towards Mn^2+^, veratryl alcohol, Reactive Black 5 or Reactive Blue 19 could be observed.

Next, laccase production was induced with 500 μm CuSO_4_ and 9 g/l ethanol, and a maximum of 540 U/L was reached in three days of culture. The enzyme was purified to homogeneity as shown by a single band between 50–75 kDa in the SDS-PAGE. The exact molecular mass (67,717 Da) and glycosylation degree (4 %) were determined by MALDI-TOF spectrometry analysis of the pure enzyme before and after deglycosylation. Laccase isolectric point was 6.2. The enzyme showed a typical UV-visible absorption spectrum for a blue laccase, with Absorbance 280 nm/Absorbance 610 nm ratio around 21 (Additional file 1: Figure S2).

The enzyme oxidized typical laccase substrates such as ABTS and 2,6-dimethoxyphenol (DMP) (Table 1), the former, with outstanding catalytic efficiency. *Lepthosphaerulina* sp. laccase showed common pH activity profiles for ABTS and DMP, with optimum activity towards ABTS at pH 3 and two peaks of activity at pH 3 and 6 for DMP oxidation (Additional file 1: Figure S3).

**Table 1:** Kinetic constants for the oxidation of ABTS and DMP by *Leptosphaerulina* sp. laccase at pH 3

	K_*m*_ (μM)	k_cat_ (s^−1^)	k_cat_/K_*m*_ (s^−1^ mM^−1^)
ABTS	16 ± 4	630 ± 47	39,899 ± 928
DMP	197 ± 50	311 ± 28	1,579 ± 214

**Fig. 1:**
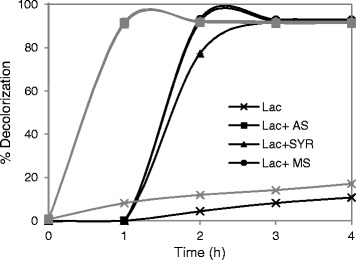
Enzymatic decolorization of synthetic organic dyes. Decolorization curves for Methyl Orange (black lines), Acid Blue 74 (grey lines) produced by *Leptosphaerulina* sp. laccase alone (−x-) and in the presence of S-type natural mediators. 50 uM dye and 100 μM redox mediator were used (AS: acetosyringone -■-; SYR: syringaldehyde-▲-; MS: methyl syringate -●-)

The enzymatic decolorization of different types of synthetic organic dyes representative of the most utilized industrial dyes (azo, anthraquinone, indigo and triarylmethane-type dyes) was evaluated by using the purified laccase alone or in the presence of redox mediators. Natural syringyl-type phenolic mediators (namely, acetosyringone, syringaldehyde and methyl syringate) and the artificial mediator 1-hydroxybenzotriazol (HBT) were tested in a molar ratio mediator/dye of 2. The enzyme was capable of decolorizing roughly 30 % of Aniline Blue and Evans Blue and 15 % of Acid Blue 74 and Methyl Orange (in four hours). These decolorization rates were significantly increased by the presence of S-type phenolic mediators, whereas HBT caused no effect (Table 2). Overall, the decolorization percentages obtained ranged from the nearly absence of decolorization of Orange II, Reactive Black 5 and Remazol Brilliant Blue by laccase alone, to the almost complete decolorization of Orange II, Methyl Orange, Evans Blue or Acid Blue 74 by laccase in the presence of natural mediators. The latter two were decolorized in 1–2 hours (Fig. 1). Over 40 % of Reactive Black 5 was decolorized by the enzyme and acetosyringone or methyl-syringate as redox mediators.

**Table 2:** Decolorization percentages for different synthetic organic dyes obtained by *Leptosphaerulina* sp. laccase (100 mU) alone or in the presence of acetosyringone (AS), syringaldehyde (SYR), methyl-syringate (MS) or HBT as redox mediators (mediator/dye ratio = 2) after 4 h of treatment

	Laccase	L-AS	L + SYR	L-MS	L-HBT
Methyl orange (azo dye)	12.8	92.2	91.5	93.1	12.0
Reactive Black 5 (di-azo dye)	3.0	41.6	17.0	41.1	2.7
Evans Blue (di azo-dye)	32.4	79.2	74.2	80.7	31.8
Orange II (azo dye)	2.9	80.0	48.2	83.0	2.6
Aniline Blue (triarylmethane dye)	29.4	47.6	37.4	47.9	30.4
Acid Blue 74 (indigo dye)	16.4	90.6	91.3	91.3	17.0
Remazol Brilliant Blue (anthraquinone dye)	2.2	20.9	56.1	15.2	1.5

By means of trypsin digestion of the purified protein and analysis of the fragmented peptides by nLC/MS-MS, the sequence of one laccase peptide could be disclosed. The peptide, (AK)WGDTIVVNVK, from *Leptosphaeria maculans* JN3 laccase (Uniprot E4ZMJ1) was found a number of times with high score. No more peptides from the pure protein could be identify in any of the searches made with other public databases, in line with the lack of results obtained from the laccase fingerprinting by MALDI TOF/TOF. Part of the sequence of the laccase peptide (AKWGDTI) was used to design a primer to amplify the laccase sequence from the fungal genome.

### Searching for laccase-type MCO genes in ***Leptosphaerulina*** sp.

**Fig. 2:**
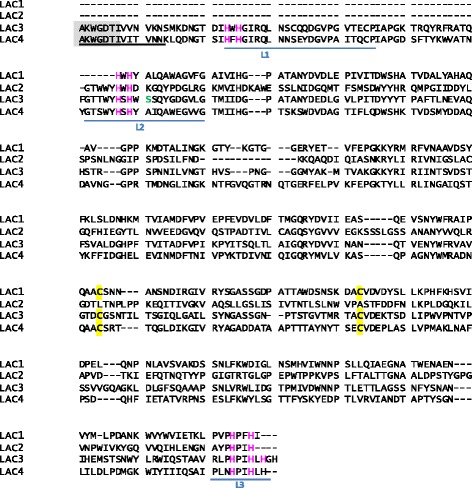
Partial protein sequences encoded by the four laccase-like genes, *lac1*, *lac2*, *lac3* and *lac4* amplified from *Leptosphaerulina sp* genomic DNA. The laccase signatures containing the copper ligands (magenta) are indicated; the peptide disclosed by proteomic analysis of the purified laccase is underlined in black, and the sequence for the corresponding degenerate peptide-based primer is highlighted in grey. Cys residues involved in one conserved S-S bridge in ascomycete laccases are highlighted in yellow

For the amplification of laccase-like genes from the genomic DNA, we designed degenerate primers based on the conserved signature sequences L2 and L3 of laccases from related fungi (L2: G T**/**S SWYHSH; L3: HP M/L/I HLHGHD F/Y/V). As a result, we amplified the partial sequences of four putative laccase genes from *Leptosphaerulina* sp. genome (*lac1, lac2, lac3* and *lac4*). The use of the aforementioned laccase peptide-based primer (AKWGDTI) allowed us to enlarge the amplification of *lac3* of and of *lac4* sequences (upstream L2 motif). However, even though *lac4* was also amplified using this primer, only Lac3 contained the exact sequence of the laccase peptide disclosed in the proteomic studies (DTIVVNVK), whereas the sequence of Lac4 was different (DTIVITVN) (Fig. 2).

The amplified partial *lac1* sequence (864pb) (GenBank KJ906511) encoded a protein that shared 58 % sequence identity with the partial sequence of *Clavariopsis quatica* laccase (GenBank ACO07312), whereas the protein encoded by *lac2* (938 pb) (GenBank KJ906512) shared 62 % sequence identity with *Stagonospora* sp. SAP143 laccase partial sequence (GenBank AAN17289). The Lac3 protein sequence, encoded by *lac3* (1074 bp) (GenBank KJ906513), was 71 % identical to the so-called similar to laccase-1 precursor from *Leptosphaeria maculans* JN3 (NCBI XP_003836225.1 = GenBank CBX92860). Finally, *lac4* (1131 bp) (GenBank KJ906514) encoded a protein with 73 % sequence identity with the hypothetical protein SNOG_06494 from *Phaeosphaeria nodorum SN15* (XP_001796864.1 = GenBank EAT86325). The four hypothetical laccases were not very similar to each other: Lac1 shared 27 % sequence identity with Lac2, 38 % with Lac3 and 41 % with Lac4.

**Fig. 3:**
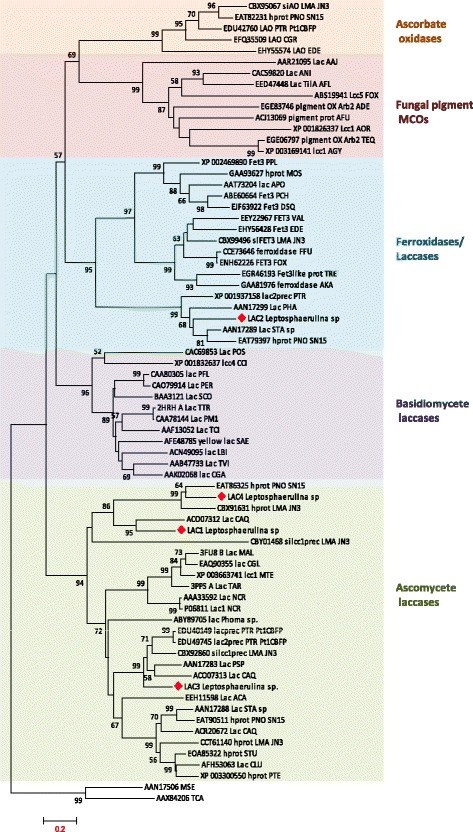
Phylogenetic tree of the four *Leptosphaerulina* sp. laccase-type proteins and selected fungal laccases, ferroxidases, ascorbate oxidases, and fungal pigment MCOs. The optimal tree constructed by the Neighbor-Joining method based on MUSCLE alignment (L2-L3 sequences length) is shown. Percentages over 50 % of replicate trees in which the associated taxa clustered together in the bootstrap test (1000 replicates) are shown next to the branches. The evolutionary distances were calculated by the JTT matrix-based method. The tree is drawn to scale, with branch lengths in the same units as those of the evolutionary distances used to infer the phylogenetic tree (number of amino acid substitutions per site). All positions containing gaps and missing data were eliminated. Evolutionary analyses were conducted in MEGA6. The GenBank accession numbers for the protein sequences are depicted together with the acronyms for the fungal strains. AAJ: *Auricularia auricula-judae*; ABE: *Arthroderma benhamia;* ACA: *Ajellomyces capsulatus*; ADE: *Ajellomyces dermatitidis*; AFL: *Aspergillus flavus*; AFU: *Aspergillus fumigatus*; AGY: *Arthroderma gypseum*; AKA: *Aspergillus kawachii*; ANI: *Aspergillus nidulans*; AOR: *Aspergillus oryzae*; APO: *Auricularia polytricha*; CAQ: *Clavariopsis aquatica*; CCI: *Coprinopsis cinerea*; CGA: *Coriolopsis gallica*; CGL: *Chaetomium globosum*; CGR: *Colletotrichum graminicola*; CLU: *Curvularia lunata*; DSQ: *Dichomitus squalens*; EDE: *Exophiala dermatitidis*; FFU: *Fusarium fujikuroi*; FOX: *Fusarium oxysporum*; LMA: *Lepthospaheria maculans*; MAL: *Melanocarpus albomyces*; MOS: *Mixia osmundae*; MTH: *Myceliophtora thermophila*; NCR: *Neurospora crassa*; PCH: *Phanerochaete chrysosporium*; PER: *Pleurotus eryngii*; PFL: *Pleurotus florida*; PNO: *Phaeosphaeria nodorum*; PSP: *Phaeosphaeria spartinicola*; PTE: *Pyrenophora teres*; PTR: *Pyrenophora tritici-repentis*; SAE: *Stropharia aeruginosa*; SCO: *Schizophyllum commune*; STA: *Stagonospora* sp.; STU: *Setosphaeria turcica*; TAR: *Theilavia arenaria*; TEQ: *Trichophyton equinum*; TCI: *Trametes cinnabarina*; TVI: *Trametes villosa*; POS: *Pleurotus ostreatus*; TRE: *Trichoderma reesei*; TTR: *Trametes trogii*; LBI: *Lacaria bicolor*; PHA: *Phaeosphaeria halima*; PPL: *Postia placenta*; VAL: *Verticillium al*falfae. The tree was rooted by two insect laccase sequences from *Manduca sexta* (MSE) and *Tribolium castaneum* (TCA)

The phylogenetic analysis of the *Leptosphaerulina sp* laccase-type MCOs built-in the sequences of known fungal MCOs belonging to different functional families: laccases *sensu stricto* (basidiomycetous and ascomycetous), fungal pigment MCOs, ferroxidases and ascorbate oxidases (Hoegger et al., 2006). MCO sequences from *Pleosporales* strains related to *Leptosphaerulina*, i.e. *Leptosphaeria maculans*, *Pyrenophora tritici-repentis*, and *Phaeosphaeria (Stagonospora) nodorum* were also included. Overall, 73 fungal sequences and two insect laccases (used to root the tree) were analyzed. The high bootstrap values confirmed robust tree topology. The phylogenetic tree showed Lac1 and Lac4 as the most related sequences among the four *Lepthosphaerulina* sp proteins. Lac1, Lac4 and Lac3, grouped in the cluster of ascomycete laccases, whereas, Lac2 clearly clustered in a separate group, together with other hypothetical laccase-like proteins, in the cluster of fungal ferroxidases (Fig. 3). No similarities with basidiomycete laccases, fungal ascorbate oxidases or fungal pigment MCOs were found, all of which gathered in differentiated clusters.

### Analysis of ***Leptosphaerulina*** sp. secretomes

Crude extracts from dye-decolorizing (7d) and laccase-induced (3d) liquid cultures were concentrated and subjected to proteomic analysis. Laccase activities in the concentrated samples were 882 U/L (1.96 U/mg of the protein content) and 1409 U/L (14.09 U/mg of the protein content), respectively. After precipitation and digestion with trypsin, the extracellular pools of proteins (EPP) were analyzed by nLC-ion trap mass spectrometry and protein homologous to predicted oxidoreductases deposited in public databases were searched.

**Fig. 4:**
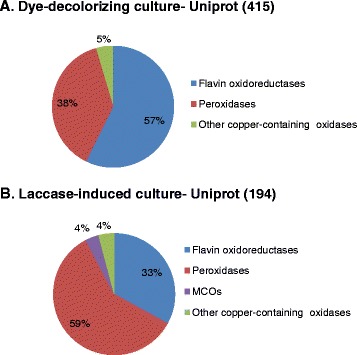
Relative abundance of the different types of oxidoreductases found in the secretomes of *Leptosphaerulina* sp. Flavin oxidoreductases (GMC oxidases, NADH oxidases and others), peroxidases (POD, DyPs, catalases), MCOs and other copper-containing oxidases from the nLC-MS-MS analysis and search against Uniprot database (restricted to Ascomycota) are depicted. **a**. dye-supplemented culture. **b**. Laccase-induced culture. The total number of oxidoreductase matches is shown in parenthesis (see Additional file 1: Tables S1 and S2 for detailed information)

Overall, homologous proteins to diverse types of oxidoreductases, peroxidases, flavo-oxidases, and copper-containing metalloproteins, were found in the shotgun analyses of both samples (Fig. 4). Flavo-oxidase like-proteins were the most abundant of the oxidoreductases found in the secretome of dye-decolorizing culture, in particular, oxidases from the Glucose-Methanol-Choline (GMC) oxidoreductase family and NADH oxidases. Most peroxidases identified by the shotgun analyses of both samples corresponded to catalase-peroxidases type. Besides, in the laccase induced culture, matching peptides from a lignin peroxidase and a dye-decolorizing peroxidase could also be found. A small percentage of copper-containing enzymes homologous to glyoxal oxidase, galactose oxidase or amine oxidase were also found in both secretomes. The most significant hits from the search against Uniprot database of the EPPs from *Leptosphaerulina* sp. grown in dye-supplemented and laccase-induced cultures are summarized in Additional file 1: Tables S1 and S2, respectively. Regarding MCOs, the aim of our study, homologous proteins could only be found in the laccase-induced sample (Fig. 4). Again, the unique peptide from *Leptosphaeria maculans* JN3 laccase (Uniprot E4ZMJ1), already found in the nLC-MS/MS analysis of the pure enzyme (AKWGDTIVVNVK), was repeatedly identified (see Additional file 1: Table S2).

Since the search against Uniprot database rendered scarce MCO hits, we focussed on the quest against the specific database LccED. More to the point, the presence in both EPPs of the four *Lepthosphaerulina* sp. laccases was investigated using a purpose-built database. The top-10 extracellular MCOs of both secretomes (identified in the searches against LccED and the in-house database) are jointly summarized in Tables 3 and 4. The protein E5Q_00271 from *Mixia osmundae* (GAA93627) was repeatedly found in both secretomes in the search against LccED. In fact, the sole MCO certainly found in the EPP from the dye-decolorizing culture was the protein homologous to E5Q 00271 (Table 3). This fungal protein presents a typical MCO sequence with the three typical cupredoxin domains containing the four conserved copper binding motifs (L1-L4). More in particular, it holds the typical constellation of acidic residues of ferroxidase sequences and it is close similar to that of *P. chrysosporium* Fe3p (ABE60664). In fact, in the phylogenetic analysis, this MCO sequence grouped with the abovementioned Fe3p and similar sequences in the ferroxidase cluster (see Fig. 2).

**Table 3:** Top-10 extracellular MCOs from the shotgun analysis of *Lepthosphaerulina* sp. secretome of the dye-decolorizing culture, identified in the searches against LccED and the *in house* database

Homologous to predicted proteins	GenBank accession number	Score	Coverage	Unique peptides	PSMs
Hypothetical [*Mixia osmundae* IAM 14324]	GAA93627	769.2	8.41	5	211
Putative multicopper oxidase [*Saccharomonospora marina* XMU15]	EHR52757	2.7	6.08	1	1
MCO type 2 (Bilirubin oxidase) [*Intrasporangium calvum*]	ADU47440	2.4	1.96	1	1
Bilirubin oxidase [*Haliangium ochraceum*]	ACY16794	2.3	1.82	1	1
Ascorbate oxidase-like [*Glycine max*]	356549511	2.1	1.22	1	1
Multicopper oxidase, putative [*Ricinus communis*]	EEF35106.1	2.0	1.17	1	1
Lac3 *Lepthosphaerulina* sp.	_	26.5	10.33	2	7

**Table 4:** Top-10 extracellular MCOs from the shotgun analysis of *Lepthosphaerulina* sp. secretome of laccase-induced culture, identified in the searches against LccED and the *in house* database

Homologous to predicted proteins	GenBank accession number	Score	Coverage	Unique peptides	PSMs
Hypothetical [*Mixia osmundae* IAM 14324]	GAA93627	1217	7.44	4	287
Similar laccase-1 precursor [*Leptosphaeria maculans* JN3]	CBX92860	842.7	2.08	2	315
Blue copper oxidase cueO precursor [*Pyrenophora tritici-repentis* Pt-1C-BFP]	EDU50675	26.4	2.40	2	8
MCO hypothetical protein [*Meyerozyma guilliermondii* ATCC 6260]	EDK39262	23.1	4.37	1	8
MCO [*Podospora anserina*]	CAP61936	12.6	1.28	1	5
*Bilirubin oxidase* precursor [*Neurospora crassa* OR74A]	EAA27114	7.0	1.38	1	3
Laccase [*Xylaria polymorpha*]	ABV32571	6.3	5.39	1	3
Lac1 *Lepthosphaerulina* sp.	_	7.0	11.90	2	2
Lac2 *Lepthosphaerulina* sp.	_	313	44.48	14	78
Lac3 *Lepthosphaerulina* sp.	_	1747	47.28	8	478

Score, sum of the scores of the individual peptides; Coverage, percentage of the protein sequence covered by identified peptides; PSM, total number of identified peptide sequences (peptide spectrum matches), including those redundantly identified

On the other hand, in the laccase-induced secretome, the search against LccED revealed the profuse finding of the same peptide from *L. maculans* laccase CBX92860 (=Uniprot E4ZMJ1) previously identified in the isolated laccase and found in Lac3 sequence (71 % identical to *L. maculans* laccase). Moreover, several unique peptides of Lac3 were recurrently found in the laccase-induced secretome using the in-house database (Table 4). Lac2 was also found in this secretome sample, and Lac1 in much lesser extent, whereas no peptides from Lac4 could be detected in the EPP from the laccase-induced culture (Table 4). On the other hand, Lac3 was the sole *Leptosphaerulina* sp. laccase identified in the dye-decolorizing secretome (Table 3). Finally, up to 11 unique peptides from Lac3 (including the peptide AKWGDTIVVNVK) were repeatedly found during the analysis of the isolated laccase when using the in-house database. Quite the opposite, not a single peptide from Lac1, Lac2 or Lac4 could be found in this sample. All these pieces of evidence confirmed Lac3 as the main laccase isoenzyme purified and characterized in this study.

## Discussion

### Lac3, a sensu-stricto laccase

The properties of the enzyme purified from the laccase-induced culture, which happened to be Lac3, strictly corresponded to the biochemical and kinetic properties of a typical fungal laccase. It is a monomeric glycoprotein, with molecular mass around 70 kDa.

Lac3 efficiently oxidizes common laccase substrates such as ABTS and DMP with high catalytic efficiency regarding other fungal laccases [17–20]. On the contrary, it was not able to oxidize HBT in an efficient manner, as shown by the similar decolorization rates obtained with or without HBT as mediator. Lac3 apparently holds a low-medium redox potential typical of most ascomycete laccases such as *Myceliophtora thermophila* laccase, which is also unable to oxidize this synthetic mediator (with redox potential above +1 V) [21]. Conversely, the oxidation of the syringyl-type phenolic compounds is facilitated by their lower redox potential (around +0.6 V) as occurred for ABTS.

Lac3 was able to decolorize several synthetic organic dyes, but Reactive Black 5 (and others) could not be directly oxidized by the enzyme mainly due to its high redox potential (about +1.3 V). Overall, the enzymatic decolorization of the dyes was notably promoted (up to 90 %) by the presence of the lignin-derived compounds, as described for basidiomycetous laccases [22]. Yet, once oxidized by the enzyme, the competence of the phenoxyl radicals of the S-type compounds to act as redox mediators depends on the chemical structure of the dye and its redox potential. Recently, it has been described the degradation of chlorophenols and chloranilines by a laccase from *Leptosphaerulina chartarum* using guiacol as redox mediator [12]. The use of natural mediators notably enhances the applicability of these low-medium-redox potential laccases as biotechnological tools in the modern biorefineries [23]. Besides, ascomycete laccases are more easily expressed than basidiomycete laccases in *Aspergillus* or yeast as heterologous hosts, being more prone to protein engineering by directed evolution [24].

### Genomic approach

The composition of MCOs in the different species might vary according to their lifestyle [25], although multiplicity events within the different type of genes frequently makes not easy to identify the connection of gene distribution to the fungal lifestyle [26]. To date, phylogenetic studies on laccases and related MCOs have revealed their grouping in functional clusters [3, 26]. In general, fungal laccases can be divided into ascomycetous and basidiomycetous clades [25] although the phylogenetic classification of some laccases does not strictly follow the species phylogeny [27, 28]. In this study, known ascomycete laccases, basidiomycete laccases, fungal ferroxidases, fungal ascorbate oxidases and fungal pigment MCOs, including MCO sequences from *Pleosporales* strains related to *Leptosphaerulina*, were used to construct the phylogeny of the four *Leptosphaerulina* MCOs. The well known laccases from white-rot basidiomycetes such as *Trametes villosa*, *Pleurotus ostreatus*, *Coriolopsis gallica*, *Trametes trogii,* etc., jointly grouped in one separate cluster. A second cluster contained characterized ascomycete laccases such as those from *Myceliphthora thermophila, Melanocarpus albomyces, Thielavia arenaria or Neurospora crassa,* together with other ascomycete hypothetical laccase sequences from the *Pleosporales* strains. Lac1, Lac3 and Lac4 from *Leptosphaerulina* sp., gathered in this cluster, thus evidencing their matching with the species phylogeny. Of the three sequences, Lac3 was the most closely related to the well characterized ascomycete laccases, suggesting it is a *sensu-stricto* laccase as it was confirmed by the biochemical characterization of the purified laccase. Moreover, Lac1, Lac3 and Lac4 partial sequences hold two Cys residues involved in one conserved S-S bridge in ascomycete laccases (C298-C332 in PDB 1GW0, *Melanocarpus albomyces* laccase) (see Fig. 3).

On the other hand, Lac2 rooted apart from laccases *sensu-stricto*, in the same node than ferroxidases, together with other hypothetical laccases from the related *Pleosporales* strains. Fungal ferroxidases such as Fet3p play a key role in iron metabolism and they are supposed to play a protective role by suppressing copper and iron cytotoxicity. Fet3 proteins hold three acidic residues, one glutamate and two aspartates (E185, D283 and D409 in *S. cerevisiae* Fet3p), near the CuT1 site which constitutes the iron binding site [29]. Besides, the disposition of negatively charged residues at the protein surface presumptively make feasible Fe^3+^ translocation to the permease Ftr1 through a pathway under electrostatic guidance [30]. When comparing the partial sequences of Lac2 or the laccase from *Phaeosphaeria halima* (AAN17299) with typical Fet3 proteins no such a collection of acidic residues could be observed. In fact, after modeling Lac2 with 1ZPU (Fet3p) as template, no iron binding site could be observed (data not shown). The position of the three acidic residues were occupied by two Asn and one Pro residues in Lac2, whereas the Tyr 354 residue, supposed to be conserved in ferroxidases [31], was replaced by Arg. The ferroxidase activity of Lac2 is therefore questioned by the lack of the typical ferroxidase residues. This cluster may meet classical (Fet3-type) ferroxidases, ferroxidases with some laccase activity - and other non *sensu-stricto* laccase-like proteins with perhaps some hybrid activities, as described previously [26].

### Proteomic approach

It is well known that laccase expression is regulated by an array of factors not merely concerned to the transcription level. The synthesis and secretion of laccases are severely influenced by the culture conditions, nutrient levels, fungal development stage and the presence of metal ions and aromatic compounds as inducers [32]. During the time course study of *Leptosphaerulina* sp. grown in liquid culture we observed an increment of laccase activity by the presence of the aromatic dye Reactive Black 5, in concordance with reported induction of laccase by organic dyes [33]. The secretion of Lac3 during dye-decolorization by the fungus was demonstrated during the shotgun analysis of the pool of proteins secreted under these conditions. However, Lac3 was not as abundant as it could be expected, in part because the time of collecting the sample (7d) did not correspond to the maximum laccase activity (3d).

Laccase production was significantly induced by the presence of Cu^2+^ and ethanol, making feasible the detection of Lac1-Lac3 in the secretome. The transcription of laccase genes is regulated by the presence of response elements in the laccase promoters such as MREs (Metal Responsive Element) and STREs (Stress responsive element) [32, 34, 35]. Besides the rapid effect of copper on the induction of transcription of laccase genes [36], Cu^2+^ might impair laccase degradation by proteases [37]. Conversely, ethanol improves laccase secretion by fungi [38] and might induce the expression of chaperones involved in protein folding and secretion [39]. The presence of different peaks of activity in time for induced (3d) and not induced cultures (5d) may suggest the occurrence of constitutive and inducible laccases in *Leptosphaerulina* sp [40–42].

Lac3, a *sensu-stricto* laccase, was the main laccase-type protein secreted by *Leptosphaerulina* sp. The role of laccases in pathogenic fungi could improve their virulence towards plants by inactivation of otherwise toxic phytoalexins or phenolics [43]. Conversely, laccase might be essential for pathogenicity by the melanization of the appressoria necessary for the penetration of the host, as described for *Colletotrichum orbiculare* (cucumber anthracnose) or *Metarhizium anisopliae* (an insect pathogenic fungi) [44, 45]. On the other hand, Lac2, a ferroxidase/laccase protein was also abundantly present in the secretome from laccase-induced culture. Peptides homologous to those from *Pyrenophora tritici-repentis* CueO were also identified in the laccase-induced sample. Besides, the ferroxidase-type protein E5Q_00257 from *Mixia osmundae* IAM 14324 was recurrently found in both dye-decolorizing and laccase-induced EPPs. This enigmatic fungus, the only species currently known in class Mixiomycetes, is an intracellular parasite of ferns (*Osmunda L*) in which it causes small yellow to brown leaf spots [46]. Iron acquisition is a critical aspect of the virulence of many pathogenic microbes. Next to its function in iron metabolism, it has been suggested a protective role for Fet3p by suppressing copper and iron cytotoxicity [47].

It is remarkable the profusion of oxidases suppliers of hydrogen peroxide in fungi. Alcohol-oxidase-type flavo-oxidases [48] and copper-containing enzymes like glyoxal oxidase or galactose oxidase were abundantly present in *Leptosphaerulina* sp. secretomes. NADH oxidases, responsible for superoxide production and cell differentiation in fungi [49] were also frequently found. All of them may play a central role in the cycling and bioavailability of metals and carbon in the natural systems. Conversely, the significant occurrence of catalase-peroxidases in plant pathogenic fungi would be driven by the need of H_2_O_2_ detoxification to deal with the host oxidative burst [50].

## Conclusions

Of the four laccase-type MCO genes found in *Leptosphaerulina* sp., three (*lac1*, *lac3* and *lac4*) encode for ascomycete laccase-like proteins, whereas Lac2 is related to ferroxidases/laccases proteins. The purification and characterization of Lac3, the main laccase produced by *Leptosphaerulina* sp. in dye decolorizing and laccase-induced cultures, allowed us to confirm that it is a *sensu-stricto* laccase with high oxidation efficiency towards ABTS and phenolic compounds. Moreover, the enzyme is able to decolorize synthetic organic dyes with high efficiency in the presence of natural phenolic mediators. These results show the way for searching for laccase-type MCOs, with possible biotechnological interest, in fungal families where the presence or function of these enzymes is poorly known. The information provided by the use of genomic and proteomic tools must be combined with classical biochemical studies since only a precise characterization of the enzyme can prove its predicted catalytic activity and confirm its potential applicability.

## Additional file

Additional file 1: Table S1. Oxidoreductases identified from the secretome of *Leptosphaerulina sp.* grown in dye-supplemented culture (7d). Only significant hits identified from the shotgun nLC/MS-MS analysis of the entire EPP after search against Uniprot Ascomycota database are shown. Protein identities provided on the basis of a single matching peptide, were considered as tentative. **Table S2.** Oxidoreductases identified from the secretome of *Leptosphaerulina sp.* grown in the laccase-induced culture (3d). Only significant hits identified from the shotgun nLC/MS-MS analysis of the entire EPP after search against Uniprot Ascomycota database are shown. Protein identities provided on the basis of a single matching peptide, were considered as tentative. **Figure S1.** Laccase (-♦-) and peroxidase (-■-) activities with ABTS detected in *Leptosphaerulina. sp*. standard liquid culture (black lines), dye-supplemented culture (red lines) and laccase-induced culture with CuSO4 and ethanol (blue lines). **Figure S2.** Biochemical characterization of the purified laccase from *Leptosphaerulina sp.* MALDI-TOF spectrometry analysis of the native and deglycosylated protein (**A**); SDS-PAGE and Coomassie Blue staining (**B**); UV-Vis absorbance spectrum (**C**). **Figure S3.** Optimum pH for oxidation of ABTS (**A**) and 2,6-dimethoxyphenol (**B)** by *Leptosphaerulina sp.* laccase. (PDF 190 kb)
