# Supplementary material for: Identification and characterization of laccase-type multicopper oxidases involved in dye-decolorization by the fungus Leptosphaerulina sp
Source: BMC Biotechnol. 2015 Aug 14;15:74. doi: 10.1186/s12896-015-0192-2 (PMC4535763; doi:10.1186/s12896-015-0192-2)
Supplement: Additional file 1: Table S1. — Oxidoreductases identified from the secretome of Leptosphaerulina sp. grown in dye-supplemented culture (7d). Only significant hits identified from the shotgun nLC/MS-MS analysis of the entire EPP after search against Uniprot Ascomycota database are shown. Protein identities provided on the basis of a single matching peptide, were considered as tentative. Table S2. Oxidoreductases identified from the secretome of Leptosphaerulina sp. grown in the laccase-induced culture (3d). Only significant hits identified from the shotgun nLC/MS-MS analysis of the entire EPP after search against Uniprot Ascomycota database are shown. Protein identities provided on the basis of a single matching peptide, were considered as tentative. Figure S1. Laccase (-♦-) and peroxidase (-■-) activities with ABTS detected in Leptosphaerulina. sp. standard liquid culture (black lines), dye-supplemented culture (red lines) and laccase-induced culture with CuSO4 and ethanol (blue lines). Figure S2. Biochemical characterization of the purified laccase from Leptosphaerulina sp. MALDI-TOF spectrometry analysis of the native and deglycosylated protein (A); SDS-PAGE and Coomassie Blue staining (B); UV-Vis absorbance spectrum (C). Figure S3. Optimum pH for oxidation of ABTS (A) and 2,6-dimethoxyphenol (B) by Leptosphaerulina sp. laccase. (PDF 190 kb) [file 12896_2015_192_MOESM1_ESM.pdf]

Supplementary Material

Identification and characterization of laccase-type multicopper oxidases involved in dye-decolorization by the fungus *Leptosphaerulina* sp.

L.S. Copete [1,2], X. Chanagá [1,2], J. Barriuso [1], M.F. López-Lucendo.[1], M.J. Martínez [1] and S. Camarero [1*]

[1]*Centro de Investigaciones Biológicas, CSIC. Ramiro de Maeztu 9, 28040 Madrid, Spain.*

[2]*PROBIOM, Universidad Nacional de Colombia Sede Medellín, Calle 59ª No 63 - 20 Medellín, Colombia*

Corresponding author: susanacam@cib.csic.es

Dr. Susana Camarero

Staff Scientist

Centro de Investigaciones Biológicas, CSIC

Ramiro de Maeztu 9, E-28040 Madrid, Spain

Phone +34 918373112, ext 4307

Table S1. Oxidoreductases identified from the secretome of *Leptosphaerulina sp.* grown in dye-supplemented culture (7d). Only significant hits identified from the shotgun nLC/MS-MS analysis of the entire EPP after search against Uniprot Ascomycota database are shown. Protein identities provided on the basis of a single matching peptide, were considered as tentative.

**Predicted proteins**	**Uniprot Accession number**	**Mw (KDa)**	**pI**	**Score**	**Proteins**	**Unique peptides**	**PSMs**	**Coverage**
**Flavin oxidoreductases (GMC oxidases, NADH oxidases and others)**								
Similar to NADH-dependent flavin oxidoreductase [*Leptosphaeria maculans* JN3]	Q0U6Y7	46.3	6.68	190.26	20	7	56	19.76
Similar to glucose-methanol-choline (GMC) oxidoreductase [*Bipolaris oryzae* ATCC 44560]	Q0UYY5	74.3	7.02	153.10	26	9	43	16.54
Similar to alcohol oxidase [*Leptosphaeria maculans* JN3]	E4ZS94	74.2	7.18	146.3	12	9	41	12.78
Alcohol oxidase p68 [*Cochliobolus victoriae*]	Q9C1S3	74.3	7.06	88.98	23	7	28	15.19
Similar to NADH-dependent flavin oxidoreductase [*Leptosphaeria maculans* JN3	E4ZXW8	47.1	6.81	58.17	1	4	17	11.40
Probable alcohol oxidase [*Fusarium fujikuroi* IMI 58289]]	G9NP24	74.6	7.14	56.23	23	4	18	5.64
NADH oxidase [*Aspergillus flavus* ATCC 200026]	B8N8Q9	44.7	7.33	53.44	39	3	19	5.08
NADH-dependent flavin oxidoreductase [*Exophiala dermatitidis* ATCC 34100]	H6C3I5	45.0	6.65	52.27	19	3	17	5.29
Similar to GMC oxidoreductase [*Leptosphaeria maculans* JN3]	Q0UII3	58.5	5.97	43.64	43	4	13	10.96
Choline oxidase [*Aspergillus flavus* ATCC 200026]	B8NYN6	60.1	6.61	32.4	19	3	9	7.93
Similar to NADH-dependent flavin oxidoreductase [*Botryotinia fuckeliana* T4)	G2XTQ3	43.7	6.64	20.24	1	1	7	3.21
Similar to Putative	J9NDG3	61.2	5.33	10.64	1	1	4	2.67

FAD-linked oxidoreductase yvdP [*Fusarium oxysporum* f. sp. *cubense* race 4]								
6-hydroxy-D-nicotine oxidase [*Arthroderma otae*]	C5FN72	62.6	6.6	9.96	1	1	3	2.56
Similar to quinone oxidoreductase [*Pyrenophora tritici-repentis* Pt-1C-BFP]	E3S510	35.8	7.14	7.95	2	1	3	4.22
Similar to GMC oxidoreductase [*Colletotrichum gloeosporioides* Cg-14]	E9F4D8	64.5	8.65	5.01	5	1	2	1.70
Similar to quinone oxidoreductase [*Pyrenophora tritici-repentis* Pt-1C-BFP	Q0U2G1	35.9	8.00	4.70	1	2	2	3.30
Similar to FAD dependent oxidoreductase [*Leptosphaeria maculans* JN3]	E4ZWP4	51.4	6.58	2.89	1	1	1	1.69
Similar to oxidoreductase [*Leptosphaeria maculans* JN3	E5A5J9	36.3	6.13	2.61	1	1	1	2.71
**Peroxidases (PODs, DyPs, catalases)**								
Catalase [*Leptosphaeria maculans* JN3]	E5ACK2	80.9	5.60	26.27	44	5	10	8.13
Similar to catalase/peroxidase [*Leptosphaeria maculans* JN3]	E5A1L6	83.1	6.06	22.80	2	2	6	2.40
Catalase [*Pyrenophora teres f. teres* 0-1]	E3RV79	80.7	6.24	18.35	44	4	7	8.24
Catalase [*Fusarium pseudograminearum*]	K3VB13	78.1	6.47	15.96	52	3	6	4.92
Catalase-peroxidase [*Pyrenophora tritici-repentis* Pt-1C-BFP]	B2WH84	84.5	6.60	12.68	3	2	4	4.08
Catalase [*Colletotrichum graminicola*]	E3Q851	77.4	6.58	8.30	13	2	3	3.36
**Other copper-containing oxidases (glyoxal oxidases, galactose oxidases, amine oxidases, etc)**								
Similar to glyoxal oxidase [*Pyrenophora tritici-repentis* Pt-1C-BFP]	Q0V6Z4	118.1	7.09	74.89	3	3	19	4,26
Similar to glyoxal	E3S209	87.0	7.23	22.91	2	2	8	3.52

oxidase [*Pyrenophora tritici-repentis* Pt-1C-BFP]								
Amine oxidase [*Phaeosphaeria nodorum* SN15]	Q0TYR0	116.6	7.2	18.15	3	3	6	3.92
Similar to peroxisomal copper amine oxidase [*Sphaerulina musiva* SO2202]	F9XI45	77.2	6.49	5.53	3	2	2	4.61
Amine oxidase [*Pyrenophora teres*]	E3RNF1	76.3	6.39	2.2	8	1	1	2.06

Score, sum of the scores of the individual peptides; Coverage, percentage of the protein sequence covered by identified peptides.; PSM, total number of identified peptide sequences (peptide spectrum matches), including those redundantly identified.

Table S2. Oxidoreductases identified from the secretome of *Leptosphaerulina sp.* grown in the laccase-induced culture (3d). Only significant hits identified from the shotgun nLC/MS-MS analysis of the entire EPP after search against Uniprot Ascomycota database are shown. Protein identities provided on the basis of a single matching peptide, were considered as tentative.

**Predicted proteins**	**Uniprot Accession number**	**Mw (KDa)**	**pI**	**Score**	**Proteins**	**Unique peptides**	**PSMs**	**Coverage**
**Flavin oxidoreductases (GMC oxidases, NADH oxidases and others)**								
Similar to GMC oxidoreductase [*Bipolaris zeicola* 26-R-13]	M2ZLK3	58.9	7.21	25.92	4	1	7	3.07
6-hydroxy-D-nicotine oxidase [*Arthroderma gypseum*]	E4UMW1	62.6	6.89	22.99	2	1	6	2.57
Similar to Polyol: NADP oxidoreductase [*Pyrenophora tritici-repentis* Pt-1C-BFP]	M2SA46	62.9	6.80	12.17	2	1	3	3.53
Similar to NADH-dependent flavin oxidoreductase [*Leptosphaeria maculans* JN3]	Q0U6Y7	46.3	6.68	19.03	34	4	6	12.94
Related to oxidoreductase [*Fusarium fujikuroi* IMI 58289]	M3B265	34.5	5.58	9.64	1	1	3	3.70
Similar to putative GMC oxidoreductase protein [*Botryotinia fuckeliana* BcDW1]	A7F2Q0	65.8	6.01	8.54	3	1	3	4.09
Similar to GMC oxidoreductase [*Marssonina brunnea* f. sp. multigermtubi MB_m1]	M3AVS4	65.1	5.83	7.11	1	2	2	2.63
Oxidoreductase	H1V3Y6	67.8	6.74	7.23	1	1	2	2.87

[*Colletotrichum higginsianum*]								
Similar to NADH oxidase, putative [*Aspergillus flavus* NRRL3357]	C7ZFN0	44.5	7.69	6.32	6	1	2	2.68
Similar to Putative FAD-linked oxidoreductase [*Fusarium oxysporum* f. sp. *cubense* race 4]	J9NDG3	61.2	5.33	6.18	1	1	2	2.67
Similar to NADH-dependent flavin oxidoreductase [*Leptosphaeria maculans* JN3]	E9DTP2	17.2	5.90	5.31	2	1	2	16.77
GMC oxidoreductase [*Leptosphaeria maculans* JN3]	E5A277	73.5	6.93	2.75	1	1	1	1.48
Similar to oxidoreductase [*Saccharomyces cerevisiae* S288c]	A7TIS1	29.2	5.77	2.73	6	1	1	4.87
**Peroxidases (PODs, DyPs, catalases)**								
Catalase [*Leptosphaeria maculans* JN3]	E5ACK2	80.9	5.6	53.73	49	4	17	6.23
Catalase [*Fusarium pseudograminearum*]	K3VB13	78.1	6.47	40.63	61	3	12	4.92
Similar to peroxidase [*Colletotrichum gloeosporioides* Cg-14], similar to putative ligninase h2 precursor protein [*Togninia minima* UCRPA7], similar to fungal lignin peroxidase [*Macrophomina phaseolina* MS6]	G2QF00	38.2	5.54	11.61	4	1	4	3.04
Dye decolorizing peroxidase [Pleurotus ostreatus]	Q0VTU1	58.2	5.86	11.50	1	3	4	7.62
**MCOs (laccases, bilirubin oxidases, CueO, ascorbate oxidases, etc)**								
Similar to laccase-1 [*Leptosphaeria maculans* JN3]	E4ZMJ1	63.5	6.13	648.6	1	1	239	1.73
Blue copper oxidase cueO [*Pyrenophora tritici-repentis*]	B2WCP7	65.8	7.44	24.01	1	2	7	2.40
Similiar to laccase-2 [*Gaeumannomyces graminis var. tritici* R3-111a-1]	M4G035	54.1	8.07	6.53	2	1	2	2.43

Similar to bilirubin oxidase [*Colletotrichum gloeosporioides* Nara gc5]	B2AES0	70.1	7.78	5.20	1	1	2	1.28
Multicopper oxidases [*Aspergillus oryzae*]	Q2UA09	66.9	5.03	4.06	1	1	2	1.33
Simililar to blue copper oxidase cueO precursor [*Pyrenophora tritici-repentis* Pt-1C-BFP]	Q0UUB4	69.8	6.76	2.97	1	1	1	1.93
**Other copper-containing oxidases (glyoxal oxidases, galactose oxidases, amine oxidases, etc)**								
Similar to glyoxal oxidase [*Pyrenophora tritici-repentis* Pt-1C-BFP]	Q0V6Z4	118.1	7.09	408.6	5	5	103	6.90
Similar to glyoxal oxidase [*Pyrenophora tritici-repentis* Pt-1C-BFP]	E3S209	87.0	7.23	77.25	3	4	23	7.03

Score, sum of the scores of the individual peptides; Coverage, percentage of the protein sequence covered by identified peptides.; PSM, total number of identified peptide sequences (peptide spectrum matches), including those redundantly identified.

Legends of supplementary figures

Fig. S1. Laccase (**-♦-**) and peroxidase (**-■-**) activities with ABTS detected in *Leptosphaerulina. sp.* standard liquid culture (black lines), dye-supplemented culture (red lines) and laccase-induced culture with CuSO4 and ethanol (blue lines).

Fig. S2. Biochemical characterization of the purified laccase from *Leptosphaerulina sp.* MALDI-TOF spectrometry analysis of the native and deglycosylated protein (**A**); SDS-PAGE and Coomassie Blue staining (**B**); UV-Vis absorbance spectrum (**C**).

Fig. S3. Optimum pH for oxidation of ABTS (**A**) and 2,6-dimethoxyphenol (**B)** by *Leptosphaerulina sp.* laccase**.**

Figure S1.

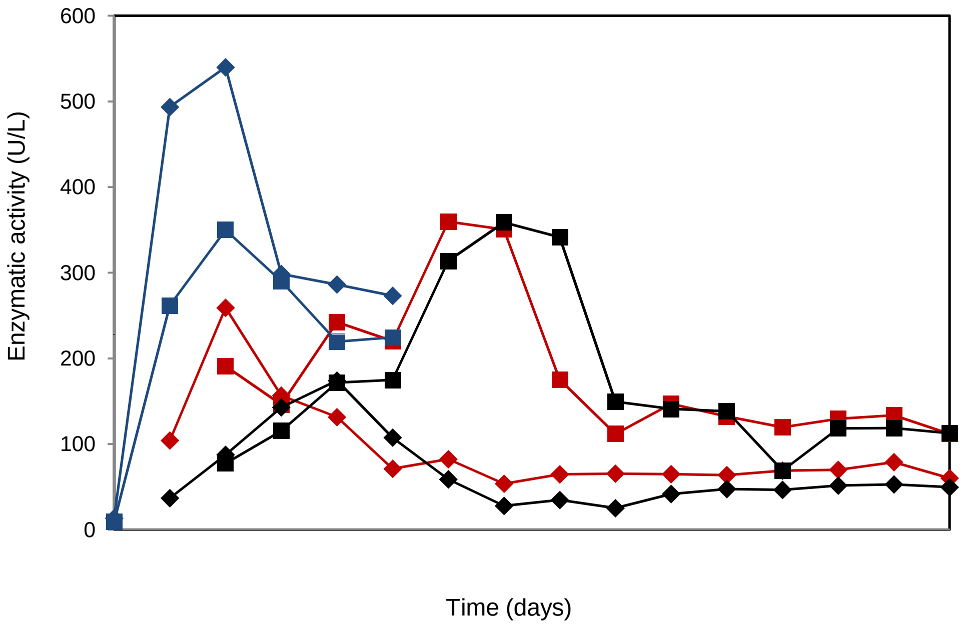

Figure S2.

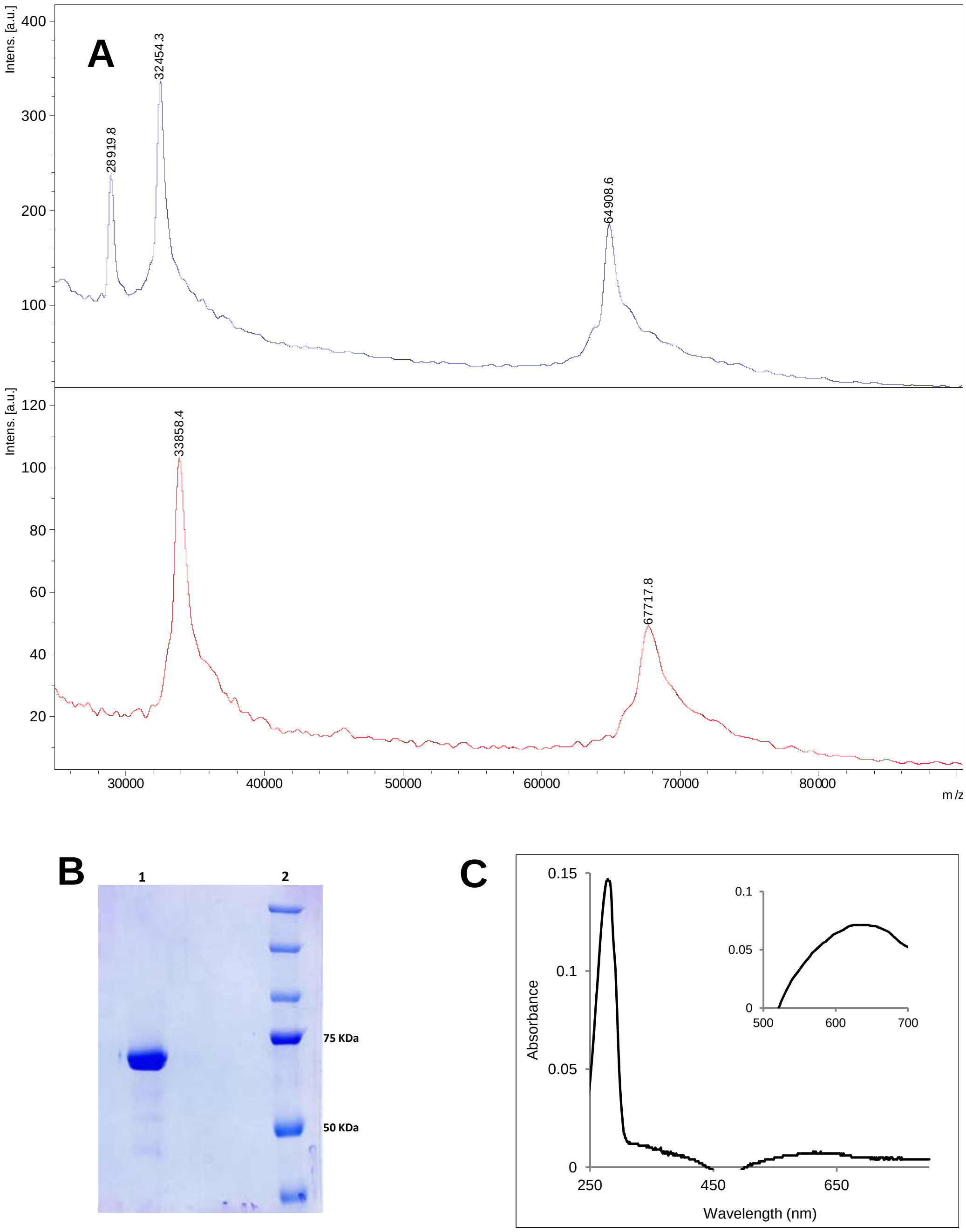
A
Intens. [a.u.]
400
300
200
100
28919.8
32454.3
64908.6
Intens. [a.u.]
120
100
80
60
40
20
33858.4
67717.8
30000
40000
50000
60000
70000
80000
m/z
B
1
2
75 KDa
50 KDa
C
0.15
0.1
0.05
0
Absorbance
250
450
650
Wavelength (nm)
0.1
0.05
0
500
600
700

Figure S3.

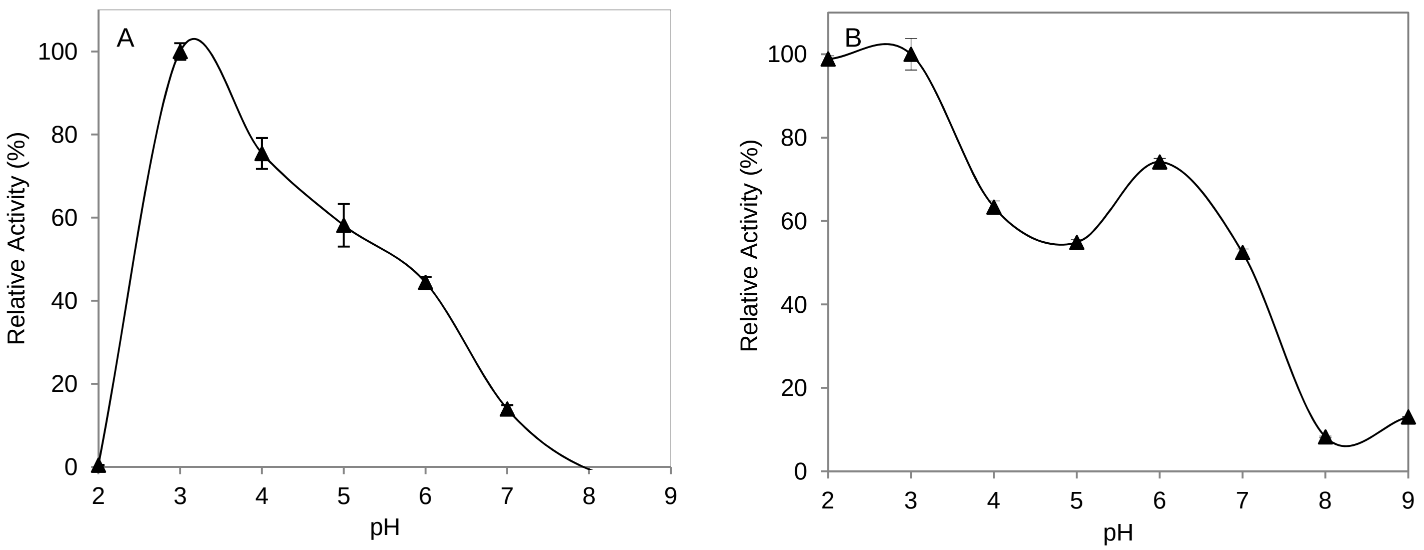
A
Relative Activity (%)
100
80
60
40
20
0
2
3
4
5
6
7
8
9
pH
B
Relative Activity (%)
100
80
60
40
20
0
2
3
4
5
6
7
8
9
pH
